# Assessing the Social and Psychological Impacts of Endemic Animal Disease Amongst Farmers

**DOI:** 10.3389/fvets.2019.00342

**Published:** 2019-10-04

**Authors:** Delyth Crimes, Gareth Enticott

**Affiliations:** School of Geography and Planning, College of Arts, Humanities and Social Sciences, Cardiff University, Cardiff, United Kingdom

**Keywords:** subjective well-being, presenteeism, social impacts, psychological impacts, animal disease, bovine Tuberculosis, farmers

## Abstract

Outbreaks of exotic animal disease, such as Foot and Mouth Disease (FMD) are associated with social and psychological impacts amongst farmers. Whilst claims of similar impacts for endemic diseases have been made, there is little empirical evidence to justify these assertions. This paper provides a descriptive analysis of the social and psychological impacts of bovine Tuberculosis (bTB) in Wales. Specifically, the paper focuses on farmers subjective well-being and presenteeism—their propensity to work suboptimally when suffering mental health problems. Results from longitudinal qualitative interviews with 16 beef and dairy farmers reveal how they derive satisfaction from their work and their emotional connection to animals, whilst the weather and red tape are most likely to affect their quality of life. Data from a postal survey (*n* = 582) using three measures of SWB, however, finds mixed evidence that animal disease is associated with farmer well-being. For all farmers surveyed, there were no significant differences in well-being between farms with and without bTB. For those farms in areas with high bTB prevalence, two of the three measures of subjective well-being showed lower levels of well-being for farmers with a history of bTB (*p* < 0.05). In conclusion, the paper discusses the policy and methodological implications for future studies of farmer well-being and animal disease.

## Introduction

Since the 2001 outbreak of Foot and Mouth Disease (FMD) in the United Kingdom, it has become widely accepted that animal disease causes emotional distress to farmers and animal keepers. These socio-psychological impacts arise from the consequences of disease management which disrupt farmers emotional bonds with their animals and the landscapes they create, and the local farming communities in which they are situated (1–9). Other studies have documented the economic impact of outbreaks of exotic disease and their subsequent effects upon farmers (10–12). Managing the mental health impacts of exotic and endemic disease outbreaks have therefore become just as important as managing animal diseases themselves. For instance, following the discovery of mycoplasma bovis in New Zealand, and an eradication plan entailing the culling of 150,000 cattle, farmers' leaders emphasized the need for mental health interventions to help farmers cope with losing their herds and potentially their businesses (13).

Whilst these accounts focus on the social and psychological impacts associated with emergency responses to exotic diseases, attempts to analyze the impacts of endemic disease are rare. On the face of it, these impacts are arguably likely to be similar: farmers may still experience a sense of loss following the slaughter of infected animals, and economic consequences may cause further mental stress. However, just as with human disease (14, 15), or other environmental risks (16, 17), the impact of endemic disease may be lessened by the familiarity with disease and the development of coping strategies to allow farmers to live with disease. Moreover, the constant presence of endemic disease calls for a broader methodological debate to help disentangle the social and psychological impacts of animal disease from those that are felt by farmers as a matter of course (18, 19), evidenced through higher levels of suicide amongst farmers than the general population (20). Whilst studies of exotic disease have employed innovative methods (21) and challenged the use of objective indicators (22) there remains a need to extend these perspectives to studies of endemic disease, whilst also testing and developing new methods of accounting for the socio-psychological impacts of animal disease.

The aim of this paper is therefore to analyze the socio-psychological impacts of an endemic cattle disease using measures of subjective well-being (broadly defined as life satisfaction) and “presenteeism” (defined as the extent and effect of attending work whilst ill). To do this, the paper draws on a survey and qualitative interviews of cattle farmers in Wales to assess the impacts of bovine Tuberculosis (bTB). The paper is structured as follows. Firstly, we provide a brief background on bTB before describing in-depth the two measures used to assess the socio-psychological impacts of the disease. Secondly, we analyze farmers' perceptions of well-being. Thirdly, we estimate levels of well-being and presenteeism amongst farmers with and without bTB. Finally, we conclude by considering the wider policy and methodological implications of these findings.

## Materials and Methods

### Background

It is frequently argued that bTB is the United Kingdom's most pressing endemic animal disease problem, resulting in the slaughter of 55,000 cattle each year at a cost of £100 million to the government (23, 24). Whilst other endemic diseases such as mastitis have a greater economic impact to cattle farmers (25), the complexities of managing bTB have the potential to cause greater socio-psychological impacts. As a statutory notifiable disease, government rather than farmers is responsible for managing bTB. However, the control of bTB is made complex by the involvement of badgers in spreading the disease, public attitudes to badger culling, scientific controversy, farmers' disease management practices, and economic incentives (26). Moreover, the statutory test and slaughter surveillance program can result in the kinds of emotional impacts experienced during FMD when cattle are killed in the wrong place or time of their lives (27). Farming organizations have contested the Government's reluctance to initiate badger culling proposals on scientific grounds, but also by highlighting the socio-psychological impacts of bTB to farmers, specifically the emotional and mental health consequences of bTB incidents (28).

The socio-psychological impacts of bTB are felt in various ways. The confirmation of a positive bTB test leads to the slaughter of infected animals. Whilst this can trigger emotional impacts to a farmer, and his/her family, the incident may also lead to economic impacts as trade is restricted. Although dairy farmers may continue to sell their milk as normal, selling store cattle or breeding stock is prohibited. The loss of milking cows will impact upon the ability to meet milk supply contracts. Herds under bTB restrictions may be allowed to restock if licenses are agreed by Government vets but negotiating a license may provide additional stress. Moreover, whilst compensation is provided for slaughtered cattle, this is unlikely to cover replacement costs or the indirect costs of additional feed for cattle that would have been sold in normal circumstances. Like FMD, an incident of bTB may also lead to a perception of stigma particularly for those farmers who rely on breeding and showing pedigree animals.

Despite these claims, there is no published academic research on the effect of bTB upon farmers' well-being or their ability to work. Some research has highlighted how farmers have become fatalistic, believing there is nothing they can do to prevent a bTB outbreak (29). Other qualitative research by farming charities concerned with farmers' welfare suggests that bTB is associated with increased levels of anxiety amongst farmers (30). However, this research is based on a self-selecting sample of farmers seeking support and offers no comparison with farmers without bTB. Other quantitative cross-sectional research finds mixed findings. For example, one study of farmers' psychiatric morbidity shows that bTB is associated with higher levels of stress than found in the general population. The duration of the bTB incident rather than the number of animals lost to the disease is associated with farmers' anxiety. Yet, whilst dairy farmers with long running bTB breakdowns experience high levels of stress, for other farm types the effects of bTB appear to be non-existent or even associated with higher levels of mental health (31).

### Approach

Previous attempts to measure farmers' mental health have relied on objective assessments, such as suicide levels (20, 32–36) or medical assessments of psychiatric morbidity as measured through standardized assessment scales (11, 34, 37–42). Other attempts have talked loosely about “farmer stress,” yet as Lobley (18) argues, stress is usually imprecisely defined. Our approach differs by measuring farmers' subjective well-being and its consequences in the form of work “presenteeism” (43). Details of each are provided below.

#### Subjective Well-Being

Subjective well-being (SWB) is derived from the difference between mental well-being (i.e., psychological functioning, life-satisfaction and ability to develop and maintain mutually benefitting relationships, personal growth, purpose in life and self-esteem) and mental illness (i.e., mental disorders affecting mood, affect and functioning) [(44), p. 2]. SWB refers to the extent to which a positive affect or emotion is felt, and the degree of life satisfaction (45). In this sense, SWB is a positive physical, social, and mental state, not just the absence of pain, discomfort and incapacity. It requires basic needs to be met, that individuals have a sense of purpose, and that they feel able to achieve important personal goals and participate in society. SWB is enhanced by conditions that include supportive personal relationships, strong and inclusive communities, good health, financial and personal security, rewarding employment, and a healthy and attractive environment [(46), p. 106].

SWB is generally acknowledged to cover two distinct dimensions (47). Firstly the *hedonic* dimension includes the subjective experience of happiness or affect and life satisfaction. Hedonic attributes of well-being are those in which the concept of happiness is primarily associated with seeking pleasure and is related to positive affect and having no worries (48). Secondly, the *eudaimonic* dimension covers positive psychological functioning, good relationships with others and self realization. The latter includes the capacity for self development, positive relations with others, autonomy, self acceptance and competence [(44), p. 7]. This second dimension is sometimes referred to as the degree of contentment an individual perceives their aspirations have been met [(49), p. 10]. Although different, these two dimensions are seen to compliment each other (48).

Measurements of SWB have been promoted as a reaction to the inability of economic measures, such as Gross Domestic Product, to fully capture a population's well-being (50). In the United Kingdom, measures of SWB have been collected at a national scale as part of the Office for National Statistics (ONS) remit to measure national well-being. Although a number of measures of SWB exist, this research employed two distinct scales. Firstly, to provide national-level comparisons, the ONS' quality of life questions were used. These ask respondents to rate their quality of life in relation to four dimensions along a 0–10 scale (question wording is shown in **Table 5**). Secondly, the validated short Warwick Edinburgh Mental Well-being Scale (SWEMWBS) was used (44, 51). This consists of seven statements against which respondents are asked to rate their feelings over the last 2 weeks on a five-point scale (see **Table 5** for question wording). These statements aim to measure a person's subjective well-being and psychological functioning using eudaimonic and hedonic perspectives. The overall score for this scale is calculated using the total scores from each statement giving a possible range of between 7 and 35. These seven statements have also been used in the UK Household Longitudinal Study and the ONS National Well-being Wheel of Measures (52) therefore allowing national level comparisons to be made.

#### Presenteeism

SWB can contribute to work absenteeism. However, it may also contribute to presenteeism—defined as the loss in productivity that occurs when employees come to work but function at less than full capacity because of ill health [(53), p. 3]. Presenteeism is particularly suited to measuring the impacts of mental health and other conditions in farming given that most farmers will be self-employed. Studies have frequently shown that farmers see “hard work” as part of their cultural identity (54), have a stoical attitude (55) and feel compelled to turn up to work whilst ill especially if the business is run single handed. Similarly, there is evidence to show that employees who have confidence in their abilities and have a high self-esteem are prone to higher levels of presenteeism particularly when workers are exposed to high physical and psychological work demands (27).

Although challenging, presenteeism can be measured using a mix of health-related measurements with assessments of productivity and economy (56–58). However, all of them make some sort of attempt to connect perceived levels of mental health with subjective or objective data of productivity (59). One such measure is the Stanford Presenteeism Scale (SPS) (60, 61). The SPS was originally developed to measure the impact of health related problems on work productivity. Presenteeism is measured through two different mechanisms. Firstly, 6 items in the SPS relate to a workers' productivity in relation to a specific health complaint. Secondly, respondents are asked to assess their usual level of productivity and their productivity when they are suffering from a health complaint (see **Table 5** for question wording). Collins et al. (62) calculate the difference between these levels of productivity and relate it to the amount respondents are paid to calculate productivity losses. Results give an overall score range between 6 and 30 with higher scores associated with higher presenteeism and self-perceived capability to focus on and complete works tasks.

### Postal Survey

Data were collected on farmers' SWB and levels of presenteeism using a postal questionnaire. The survey included the aforementioned SWB (ONS and SWEMWBS) and presenteeism (SPS) scales. In addition, the survey collected data on respondent characteristics, farm type and management, pressures facing farmers, and attitudes toward bTB policy. Attitudinal questions used 1–5 scales ranging from strongly disagree to strongly agree. Respondents were also able to add additional comments in free-text boxes.

A postal questionnaire was distributed to 1,800 farmers in Wales in May/June 2013. Respondents were randomly sampled from the Animal and Plant Health Agency's bTB database. The sampling frame was based on a quota sample of 600 each of dairy and beef cattle farmers across Wales, and a randomly selected group of 600 farms that were experiencing a bTB incident at the time survey. The survey was printed in both English and Welsh languages with 8% completing in Welsh which were translated by a native Welsh speaker. The overall response rate was 33% (*n* = 582) and the specific areas of Wales that generated the highest proportion of responses were: Carmarthenshire (18% of all responses), Pembrokeshire (17%), Mid Powys (12%), and Gwent (10%). Respondents were supplied with a pre-paid return envelope and a reminder was sent to participants 3 weeks into the survey period to encourage response.

Given that bTB and mental well-being feature in attempts to influence Government policy, it was important to minimize the potential for social desirability bias. The survey was therefore described as an attempt to understand farmers' well-being, rather than explicitly the effect of bTB on farmers' well-being. Any questions relating directly to bTB were placed at the end of the questionnaire after the questions on well-being to minimize bias. In addition, the SPS productivity questions were adapted to exclude direct reference to bTB in order to minimize bias and allow comparison with bTB-free farms.

### Qualitative Interviews

To explore in more depth the socio-psychological impact of bTB, 16 farmers were interviewed three times over 18 months. Respondents were based in four areas of Wales with the highest bTB prevalence. Farmers were identified with the assistance of local vets in each area who recommended potential farmers from their knowledge of bTB incidents. Those that agreed to participate were asked about the meaning of well-being with farmers, the socio-psychological impacts of bTB and were observed during bTB-related events on the farm, such as bTB testing. To ensure confidentiality, all names of respondents have been removed and areas coded alphabetically. Characteristics of each respondent are shown in Table 1. Further methodological details are found in Crimes (63).

**Table 1:** Breakdown of farm characteristics for farmer participants.

**Farm**	**Farm enterprises**	**Herd size**	**Farm size (acres)**	**bTB status Oct 2012^a^**	**bTB status April 2014^a^**
Beef farm, area 1	Beef	19	108	Free	Free
Dairy farm, area 1	Dairy and beef	240	250	Free	Restricted
Beef and sheep farm, area 1	Suckler beef and sheep	16	140	Free	Free
Dairy and sheep farm, area 1	Dairy and sheep	200	350	Free	Restricted
Dairy farm a, area 2	Dairy only	325	140	Restricted	Restricted
Dairy farm b, area 2	Dairy only	100	225	Free	Free
Dairy farm c, area 2	Dairy only	160	350	Restricted	Free
Dairy farm d, area 2	Dairy only	220	200	Restricted	Free
Dairy and beef farm, area 2	Dairy and beef	300	400	Restricted	Free
Beef farm, area 3	Suckler beef	61	138	Restricted	Restricted
Beef and sheep farm, area 3	Suckler beef and sheep	160	tbc	Restricted	Free
Dairy farm, area 3	Dairy and beef	486	400	Restricted	Free
Beef and sheep farm, area 4	Suckler beef and sheep	60	277	Restricted	Free
Dairy and beef farm, area 4	Dairy, beef and sheep	600	500	Restricted	Free
Beef and arable farm, area 4	Beef and arable	160	500	Free	Free
Dairy farm, area 4	Dairy only	120	250	Restricted	Free

a *Refers to bTB status of each farm at the start and end of the longitudinal interviews. Farms with bTB are restricted; farms without are free*.

### Analysis

Survey data were coded and entered into SPSS. Coding followed the instructions relevant to the SWEMWBS and SPS. To test for significant differences between farm types and bTB status, bivariate significance tests (independent samples *t*-tests) were used. Multiple response questions were coded into discrete categories. For example, questions asking about farm disease threats were coded into six main categories reflecting different disease types. These included: diseases that affected the fertility of cattle (e.g., IBR, BVD); bovine Tuberculosis; diseases that affect the productivity of cattle (e.g., mastitis); diseases that affected youngstock (e.g., scour, pneumonia); other cattle diseases; diseases that affected other animals on the farm (e.g., orf in sheep); or none. Each respondent had five opportunities to identify and rank a disease and results from the survey show that in total, 1,680 disease threats were cited (**Table 3**). A total of 170 farmers (32%) wrote down the maximum of five diseases. Interviews were fully transcribed and analyzed using QSR Nvivo 8 software. Coding was carried out using an inductive approach by initially keeping a record of codes that emerged during transcription alongside those which were anticipated from the existing literature.

## Analysis

### Respondent Characteristics

Table 2 shows the farm types and geographical locations of surveyed farmers. The average number of cattle on holdings from the survey was 166 with a range of between 1 and 1,400 cattle. Geographically, most responses came from dairy farms in from Carmarthenshire (26.7%) and Pembrokeshire (24.8%). Seventy percentage of the farmers stated they were owner occupiers, 14% were tenanted and 15% of mixed tenure. Respondents tended to be elderly farmers: 33% were aged 55–64, and 24% over 65. Just 0.5% were under 25. This distribution reflects the average age of all farmers in Wales of 55 (64). Most (89%) responses were from male farmers.

**Table 2:** Proportion of farms by main farm type and geographical area.

**Main farm type/Area**	**Dairy (%)**	**Suckler beef (%)**	**Beef stores (%)**	**Beef finishing (%)**	**Arable (%)**	**Sheep (%)**	**Other (%)**	**All %**
All Wales	37	37	9	5	<1	10	2	–
Anglesey	1.0	3.4	6.2	7.7	–	1.8	15.4	3.1
Gwynedd	6.7	8.7	6.2	3.8	–	3.6	–	6.7
Clwyd	11.0	5.8	12.5	23.1	–	9.1	7.7	10.5
Ceredigion	8.6	10.1	8.3	–	–	9.1	15.4	8.9
Pembrokeshire	24.8	13.0	12.5	15.4	–	7.3	23.1	17.4
Carmarthenshire	26.7	13.0	16.7	11.5	–	12.7	15.4	18.1
North Powys	5.2	2.9	4.2	3.8	–	9.1	–	4.5
Mid Powys	6.7	17.4	8.3	7.7	–	20.0	7.7	11.9
South Powys	–	3.9	-	3.8	–	7.3	–	2.4
South Glamorgan	1.9	1.4	6.2	3.8	–	–	–	2.0
Mid Glamorgan	1.9	1.4	2.1	3.8	–	–	7.7	1.8
West Glamorgan	–	5.8	2.1	3.8	–	–	–	2.5
Gwent	5.7	12.1	12.5	11.5	100	16.4	7.7	10.3

Twenty one percentage of farms were under bTB restrictions at the time of the survey. Of the remainder, 41% stated that their farms had never been under bTB restriction with 59% stating that they had been under bTB restrictions in the past. The most frequently cited disease threat was bTB: three-quarters (74%) of respondents mentioned this disease as a threat, and it accounted for 26% of all disease mentions. As respondents frequently had more than one enterprise on the farm, diseases of other animals such as sheep, pigs or poultry were also mentioned (Table 3).

**Table 3:** Diseases cited as most significant by surveyed farmers.

**Disease concern**	**Primary disease concern**		**Top 5 disease concerns**			**Primary disease concerns**					
						**Under bTB restriction**		**Never had bTB**		**Area bTB Prevalence**	
	***N***	**Respondents (%)**	***N***	**All cases (%)**	**All respondents (%)**	**Yes (%)**	**No (%)**	**Never had bTB (%)**	**Have had bTB (%)**	**Low/medium (%)**	**High risk (%)**
Fertility	29	5.0	227	13.5	39.0	0.9	6.3	4.8	7.9	7.3	3.8
bTB	283	48.6	433	25.8	74.4	84.2	42.4	20.5	60.0	33.7	59.3
Production diseases	57	9.8	287	17.1	49.3	3.5	11.8	14.5	10.3	15.5	7.4
Youngstock diseases	28	4.8	146	8.7	25.1	1.8	5.7	5.4	5.4	7.3	3.8
Other cattle diseases	8	1.4	83	4.9	14.3	0.0	1.8	4.8	0.0	2.0	1.1
Other animal diseases	96	16.5	411	24.5	70.6	6.1	19.5	30.1	11.6	19.2	15.4
No diseases	81	13.9	93	5.5	16.0	3.5	12.5	19.9	4.5	15.0	9.1

### Farmers Understandings of Well-Being

When farmers were asked to think about the definition of well-being, what it meant to them and what they thought influenced both positive and negative levels of well-being, analysis of the interviews revealed a number of distinct themes in farmers' understandings of well-being. They are as follows:

#### Well-Being as Health

The main association farmers drew with the concept of well-being was with the idea of health. Farmers distinguished between good physical and mental health as being a sign of well-being. In relation to physical health, farmers commented that being fit and able to work hard was a sign of well-being. For example:

“*I would say well-being to me is being fit and healthy and probably able to do what you want to a reasonable extent. That's how I would describe it.” (Dairy and beef farmer, area 4)*.

Closely related to physical health was mental health. One farming couple believed that their mental well-being was not a problem because of the enjoyment they received from farming and its way of life. This farmer compared how farming had kept him physically fit compared to others of his age who he had been at school with who had pursued other careers.

#### Happiness

A second theme was of happiness which connects to the much broader concept of eudaimonia identified in the well-being literature rather than just the physical and mental aspects of health. Several farmers commented that this sense of happiness or contentment came directly from their work even though they were probably working harder than they should. Happiness also stemmed from farmers' work with animals and the sense of pride and enjoyment they got from that. This was particularly the case in relation to pride in farmers stock and being able to complete the cycle of birth to death, or demonstrate success in front of fellow farmers:

“*But I suppose the real joy out of it comes when you're selling something for, you know, good money or taking it to a show and winning or seeing that you've done something with it, like getting a bull into AI or something like that.” (dairy and beef farmer, area 4)*.

#### Freedom and Individuality

Connected to the idea of respect, some farmers also commented that being able to get on to do their job was inherently related to well-being. For example:

“*I don't complain about my quality of life, because I'm quite happy, I love my cows, I love my calves, so long as everybody leaves me alone I'm quite happy.” (Dairy farmers a, area 2)*.

Similar ideas are expressed in ideas of good farming (65) in which the bureaucracy of farming is seen in a negative light. However, this sense of loneliness contrasts with other theories of well-being which suggests that communal and social connectivity are important:

“*A good network of support and friendship and that sort of thing as well which contributes to a feeling of well-being I would of thought and rather than if you stayed slogging your guts out all day and you don't go through the farm gate you get, you feel probably depressed and down, you get stressed and what have you and you need to have that external factors that help you with feeling good about yourself and life and what you're doing I suppose isn't it I would think” (dairy farmer e, area 2)*.

#### Natural Living

Farming is a way of life rather than just an occupation. A comparison is made here to a pleasurable working environment with the example provided below of working in a city or those in other occupations. One farmer felt that there were moments in his working day when he would feel that the location of where he worked counteracted the dull routine jobs particularly during the winter months.

“*It's just been the usual mundane at the moment but there are, I think, now and again a moment or something, it depends where you are on the farm, you look around and you think it's a nice place to be anyway, we've got a few nice spots on the farm where we can view right up to Preselis and so forth and you think it's not a bad spot to be in you know what I mean” (dairy farmer e, area 2)*.

### Farming Pressures

Farmers were asked to rank in order of importance five farming pressures. Seven broad themes emerged from the coding: finance, the weather, bovine TB, red tape/bureaucracy, paperwork, farm management, and other factors (see Table 4). It was clear from the interviews, however, that some of these pressures were inter-connected and affected different farmers in different ways.

**Table 4:** Farming Pressures cited as most significant by surveyed farmers.

**Farming pressure**	**Primary farming pressure**		**Top 5 farming pressures**			**Primary farming pressure**					
						**Under bTB restriction**		**Never had bTB**		**Area bTB Prevalence**	
	***N***	**Respondents (%)**	***N***	**All cases (%)**	**All respondents (%)**	**Yes (%)**	**No (%)**	**Never had bTB (%)**	**Have had bTB (%)**	**Low/ medium (%)**	**High risk (%)**
Finance	153	27.70	550	23.00	99.50	26.50	27.80	34.80	24.60	30.70	25.80
Weather	100	18.10	390	16.30	70.50	14.20	19.20	18.60	19.20	19.00	18.00
TB	96	17.40	273	11.40	49.40	31.00	13.90	8.70	18.30	11.60	20.50
Paperwork	78	14.10	256	10.70	46.30	8.80	15.30	13.00	14.60	14.80	14.00
Red tape	46	8.30	301	12.60	54.40	7.10	8.80	9.30	7.90	7.40	8.40
Farm management	72	13.00	440	18.40	79.60	12.40	13.40	13.00	14.20	14.80	12.10
Other	7	1.30	177	7.40	32.00	0.00	1.60	2.50	1.30	1.60	1.10

#### Weather

Weather was cited as the most significant amount of stress by over 70% of farmers. Extreme wind and rain or lack of rain, could all make farming more stressful, placing pressure on budgets by restricting the supply of animal feed. More broadly, farmers connected the weather to seasonality. The seasons influence the kinds of farming activities that are possible, and in doing so impacting upon well-being:

“*I look forward to the cows going out and the grass is growing like hell and I know that the winter chores are coming to an end, because it's just the monotony of scraping out and feeding every day; how these people keep their cows in all year round I don't know because I'm quite happy to see them go out, that's why it's been a pain in the backside the last two summers” (Dairy farmer e, area 2)*.

“*To me, seeing a calf or a lamb born, seeing new life. I've always been…looked forward to lambing time because the time of year is nice and the days are longer and…I don't know what it is, I don't know, but seeing lambs born and helping them, pulling them whatever, it just gives some…it's a great pleasure.” (beef and sheep farmers, area 3, Welsh Translation)*.

These preferences were individual: some farmers preferred preferred the routine of working during the winter time as the summer months could be very busy times. Harvesting could take up long hours during the day and also was dependable on climatic conditions.

#### Red Tape and Bureaucracy

Paperwork, form filling and the increased hours farmers have to spend on these tasks rather than physically farming were cited as key influences upon well-being. Whilst this pervaded all aspects of farming, it was also reflected in farmers concerns about the politics of farming and in the Welsh Governments handling of bTB. To demonstrate, one farmer produced a volume of paperwork during the interview:

“*Let me just show you something a minute. That is the total paperwork for one reactor and one doubtful [IR]. Pages and pages [pages rustling]. There is no sense in the amount of paperwork that they [Government] churn out.” (Beef farmer, area 4)*.

#### Finance

Financial pressures were connected to the restrictions the weather and bTB placed on the way farmers could manage their farm. For example, the weather affected business profitability due to poor milk prices and higher feed costs. An outbreak of bTB could lead to overstocking requiring more work and employing additional staff to help with the workload. Feed, fertilizer and fuel bills were elevated with more cattle to feed and the lack of income from being unable to sell surplus livestock such as heifers for breeding or store beef cattle unless they went directly to slaughter.

Whilst these impacts may appear to play into the cultural identity of “hard work,” they also limited other aspects of “good farming.” For example, in describing what they enjoyed about farming, farmers referred to their contact with animals, participating in agricultural social events, and maintaining a tidy farm:

“*I like to see a tidy farm, I like to see the fields in a nice state and that's really not happening at the moment, I like things to be tidy, the hedges aren't cut, there's ruts in the field…I like improving the farm, I like improving the farm we have done that quite a bit as well, I like putting down concrete and make the road look nice.” (dairy farmer a, area 2)*.

Whilst this cultural ideal of farming was therefore a strong influence in farmers' well-being, either through regulation, a lack of time or money, pressures such as bTB could prevent these culturally symbolic aspects of farming from happening:

“*What they get frustrated about is that [my children] like showing calves, and they have their favourite calves, that they cannot take them because they're not allowed to go from here. So this year we had to buy them two calves to the other farm, but that means that they have to go every day to the other farm to train them which is five miles away. So it's nowhere near as convenient as doing it here. But the danger with bTB is that it could take their favourite calves away, but I suppose that's part of life. They have to get used to it.” (dairy and beef farmer, area 4)*.

### Farmers' Well-Being

Farmers perceived SWB and levels of presenteeism and productivity are shown in Tables 5–**7**. For the ONS measures, farmers' perceived SWB was lower than for the general population in the UK and Wales as judged by the Annual Population Survey (21), as well as analagous “skilled trades” occupations which include farmers (66). Measures of SWB included in the SWEMWBS were also scored lower by farmers than other surveys using the same measures (67). Farmers score higher in the eudaimonc well-being components than the hedonic component. This is also a trend in both the national population and occupational group scores.

**Table 5:** SWB Questions, response scales, and mean scores.

**Scale**	**Question**	**Response scale**	**Mean response**
ONS	Overall how satisfied are you with your life nowadays?	0–10 (“not at all satisfied”—“completely satisfied”)	6.51
	Overall to what extent do you feel the things you do in your life are worthwhile?	0–10 (“not at all worthwhile”—“completely worthwhile”)	6.82
	Overall how happy did you feel yesterday?	0–10 (“not at all happy”—“completely happy”)	6.90
	Overall how anxious did you feel yesterday?^a^	0–10 (“not at all anxious”—“completely anxious”)	4.45
SWEWBMS	I've been feeling optimistic about the future	1–5 (Strongly disagree—Strongly agree)	3.03
	I've been feeling useful		3.53
	I've been feeling relaxed		2.88
	I've been dealing with problems well		3.52
	I've been thinking clearly		3.63
	I've been feeling close to other people		3.37
	I've been able to make up my own mind about things		3.98
SPS	The stresses of my job were much harder to handle^a^	1–5 (Strongly disagree—Strongly agree)	3.35
	I was able to finish hard tasks in my work		3.59
	I've been distracted from taking pleasure in my work^a^		3.27
	I felt hopeless about finishing certain work tasks^a^		2.75
	At work, I was able to focus on achieving my goals		3.45
	I felt energetic enough to complete all my work		3.14
SPS productivity	In the last month the % of my work time that I was as productive as usual was:	Percentage of time	75.62
	Compared to my usual level of productivity, in the last month the % of my work that I was able to accomplish was:		75.22
	In the last month, the % of my work time that I was likely to make more mistakes than usual was:		26.30
	**Total ONS score**		25.90
	**Total SWEMWBS score**		23.92
	**Total SPS score**		18.78

a *Scales are reversed when calculating combined scores*.

Farmers' perceptions of presenteeism from the SPS give an overall mean score of 18.78 for respondents to the farmer survey out of a possible range of between 6 and 30. In terms of perceived productivity (**Table 7**), farmers reported that in the past month they were 75.6% as productive as usual (i.e., a 24.4% loss in productivity compared to their usual level). Farmers were able to accomplish 75.3% of their work compared to their usual level of productivity (i.e., almost 25% of their work was unaccomplished). They also felt that they were likely to make more mistakes than usual in their work time (26.3%).

### Farm Level Characteristics and Well-Being

**Table 7** compares farmers' perceptions of well-being with their personal characteristics. Farmers over 65 were significantly likely to have lower well-being (*p* < 0.05) than young age groups as judged through the ONS and SPS scales. Well-being did not vary significantly between gender.

Differences in farm characteristics and SWB are not statistically significant. For the ONS and SWEMWBS measures, beef farms had the highest levels of perceived well-being and dairy farmers the lowest (see Tables 6, 7). The highest presenteeism scores are displayed by beef finishing and beef stores farm types. Work productivity scores of dairy farmers are highest. Well-being also varied by herd size (see Table 6) with smaller herds recording higher levels of well-being. Small dairy herds also had higher levels of well-being whilst dairy farmers with herd sizes 251–500 display better work productivity than for other combination of enterprises and herd sizes.

**Table 6:** Analysis of farmer well-being and work productivity mean scores by herd size and main farm type.

	**Farm type**	**1–50**	**51–150**	**151–250**	**251–500**	**501+**
ONS	Dairy	26.67	25.72	26.07	24.37	24.87
	Suckler Beef	26.85	24.73	21.74	22.29	33.00
	Beef Finishing/Stores	28.66	25.96	26.60	27.00	24.00
WEMWBS	Dairy	22.44	24.06	23.98	23.94	24.39
	Suckler Beef	24.38	23.37	23.12	23.86	23.00
	Beef Finishing/Stores	26.31	24.12	23.00	22.33	27.00
SPS	Dairy	18.78	19.31	19.05	18.12	18.42
	Suckler Beef	18.38	18.53	17.18	17.00	23.00
	Beef Finishing/Stores	19.94	18.88	19.50	20.00	17.00
WP1	Dairy	72.78	80.07	78.14	79.18	77.24
	Suckler Beef	71.55	74.59	72.47	67.86	100.00
	Beef Finishing/Stores	76.34	77.29	73.50	80.00	50.00
WP2	Dairy	71.11	78.80	77.69	81.04	79.14
	Suckler Beef	72.81	71.08	68.47	77.14	100.00
	Beef Finishing/Stores	74.84	77.42	76.50	83.33	50.00
WP3	Dairy	14.44	22.75	27.98	21.79	24.29
	Suckler Beef	27.80	28.40	15.25	44.29	10.00
	Beef Finishing/Stores	28.03	22.75	29.00	33.33	70.00

*Source: survey data*.

*Mean scores are provided*.

*Sample sizes (n): Dairy−201, Sucker beef−207, Beef finishing/stores−75, Other/missing−71*.

*WP1, Work productivity 1 (in the last month, the % of my time I was as productive as usual was:)*.

*WP2, Work productivity 2 (compared to my usual level of productivity, in the last month the % of my work that I was able to accomplish was:)*.

*WP3, Work productivity 3-(in the last month, the % of my work time that I was likely to make more mistakes than usual was:)*.

**Table 7:** Mean SWB ratings and farm characteristics.

		**ONS mean**	**SWEWBMS mean**	**SPS mean**	**WP1 mean %**	**WP2 mean %**	**WP3 mean %**
All respondents	All Farms	25.90	23.92	18.78	75.62	75.22	26.30
	bTB Free when surveyed	26.13	23.84	18.96	75.83	75.66	26.01
	Farms with bTB when surveyed	24.80	24.08	18.14	75.54	74.28	26.33
	Never had bTB	26.75	24.21	19.26	75.87	74.33	27.55
Low risk areas	All Farms	25.84	23.24	18.62	73.44	74.28	26.59
	bTB Free when surveyed	25.70	23.22	18.54	72.84	73.67	27.86
	Farms with bTB when surveyed	28.64	24.00	20.44	87.40	88.30	9.70
	Never had bTB	25.77	23.42	18.91	72.99	73.34	27.72
High risk areas	All Farms	25.88	24.15	18.86	76.33	75.86	26.02
	bTB Free when surveyed	26.26	24.12	19.16	77.16	76.92	25.00
	Farms with bTB when surveyed	24.40	24.07	17.94	74.03	72.92	28.00
	Never had bTB	27.59	24.91	19.76	79.01	76.88	26.61
Farm characteristics	Dairy	25.31	23.91	18.75	78.47	78.7	23.89
	Suckler beef	25.41	23.76	18.33	73.05	72.12	27.4
	Beef finishing	27.4	24.97	19.55	76.15	76.36	27.13
Farmer characteristics	Under 35	26.74	24.11	18.83	84.41	81.18	24.53
	35–44	26.45	24.51	19.16	80.60	82.20	29.11
	45–54	24.73	23.28	18.21	75.53	74.51	28.35
	55–64	24.90	23.37	18.29	73.49	73.67	23.66
	>65	27.44	25.09	19.28	74.88	74.43	25.73
	Male	25.65	23.95	18.65	75.62	75.52	26.21
	Female	25.71	23.32	18.06	75.37	74.23	26.73

Farmers who identified finance as a key pressure were statistically significantly more likely to have lower well-being levels for all three measures. These farmers were also more likely to report reductions in the time that they were productive. Farmers reporting red tape as a key pressure were also statistically significantly likely to have lower well-being scores across all three well-being measures.

### Bovine Tuberculosis and Farmers' Well-Being

No clear differences were detected between well-being levels and farm bTB status. For the ONS and SPS scales, well-being was marginally higher for bTB-free farms. Results for the SWEMWBS revealed well-being was lower for bTB-free farms. Farmers with bTB were marginally less productive than those without bTB. However, none of these differences were statistically significant, and the small differences suggest there is little difference between farmers' well-being and productivity with and without bTB (Table 7).

Further analysis compared responses to individual questions in each of the well-being scales to see if there were differences between the individual well-being domains and bTB. On the ONS scale, farmers with bTB were less likely to say that their life was worthwhile (*p* = 0.046), but none of the SWEMWBS measures were significantly different. For the SPS, farmers with bTB were more likely to report that the stresses of their job were hard to handle than those without bTB (*p* = 0.02).

For all three measures of SWB, farmers reporting bTB as a pressure were more likely to report higher levels of well-being, although these differences are not statistically significant. Levels of SWB were not lower amongst farmers who had a bTB test in the next month. Neither were dairy farmers or larger herds with a bTB incident more likely to have higher or lower levels of SWB or presenteeism than those that were bTB free.

There are no statistically significant differences between measures of SWB or presenteeism between farmers in areas of different bTB prevalence. However, farmers with bTB in high-risk areas, however, do have statistically significantly lower levels of well-being than those without (Table 7). For the ONS measures, the mean level of well-being is 24.40 for farmers with bTB compared to 26.26 on bTB free farms (*p* = 0.041). SWEMWBS scores differed by 0.05 and was not statistically significant. Farmers' SPS score on bTB affected farms was 17.94 compared to 19.16 on bTB free farms (*p* = 0.022). Productivity scores were higher for bTB farms, although these differences were not statistically significant. For farms that have never had bTB, both well-being and productivity scores were higher than for those farms with a bTB history. However, none of these differences were statistically significant whether analysis was conducted for all farms, or separately for those in low and high-risk areas.

Measures of SWB were significantly correlated with estimates of productivity for all respondents, and in the low and high-risk areas (Table 8). For all respondents the correlations between productivity and SPS measures were highest for farms with bTB when surveyed (*r* = +0.694) for WP2. In other words, farmers with higher levels of SWB were also the most productive. For the same measure, the correlation was lower for bTB-free farms (*r* = +0.489) and farms that had never had bTB (r = +0.543), indicating lower productivity. This trend was also evident for correlations between productivity measures and the ONS and SWEWBMS. In low risk areas, these correlations show no consistent pattern. In high-risk areas, correlations are stronger for farms with bTB. For example, WP2 is strongly correlated with SPS on farms with bTB when surveyed (*r* = +0.706) whilst the correlation for bTB free farms was weaker (*r* = 0.487). For all measures of SWB in high-risk areas, correlations of SWB and productivity were higher for farms with bTB.

**Table 8:** Correlation co-efficients between subjective well-being and work productivity measures.

		**ONS**			**SWEWBMS**			**SPS**		
		**WP1**	**WP2**	**WP3**	**WP1**	**WP2**	**WP3**	**WP1**	**WP2**	**WP3**
All respondents	All Farms	0.489**	0.437**	−0.331**	0.513**	0.466**	−0.277**	0.553**	0.540**	−0.392**
	bTB Free when surveyed	0.442**	0.378**	−0.290**	0.488**	0.423**	−0.265**	0.527**	0.489**	−0.343**
	Farms with bTB when surveyed	0.647**	0.634**	−0.490**	0.603**	0.616**	−0.414**	0.619**	0.694**	−0.558**
	Never had bTB	0.463**	0.390**	−0.414**	0.510**	0.420**	−0.340**	0.594**	0.543**	−0.390**
Low risk areas	All Farms	0.439**	0.334**	−0.331**	0.554**	0.458**	−0.378**	0.563**	0.486**	−0.444**
	bTB Free when surveyed	0.395**	0.292**	−0.314**	0.548**	0.440**	−0.380**	0.547**	0.463**	−0.421**
	Farms with bTB when surveyed	0.695**	0.517*	−0.406	0.385	0.406	−0.093	0.581*	0.543*	−0.624*
	Never had bTB	0.364**	0.312**	−0.438**	0.507**	0.444**	−0.477**	0.554**	0.505**	−0.436**
High risk areas	All Farms	0.512**	0.508**	−0.336**	0.498**	0.480**	−0.246**	0.538**	0.559**	−0.376**
	bTB Free when surveyed	0.463**	0.457**	−0.279**	0.451**	0.422**	−0.203**	0.494**	0.487**	−0.302**
	Farms with bTB when surveyed	0.640**	0.643**	−0.501**	0.625**	0.630**	−0.451**	0.621**	0.706**	−0.557**
	Never had bTB	0.549**	0.552**	−0.408**	0.504**	0.414**	−0.231	0.594**	0.538**	−0.367**

* *Correlation is significant at the 0.05 level (2-tailed)*.

** *Correlation is significant at the 0.01 level (2-tailed)*.

*WP1, Work productivity 1 (in the last month, the % of my time I was as productive as usual was:)*.

*WP2, Work productivity 2 (compared to my usual level of productivity, in the last month the % of my work that I was able to accomplish was:)*.

*WP3, Work productivity 3-(in the last month, the % of my work time that I was likely to make more mistakes than usual was:)*.

## Discussion

These results are, in some senses, unexpected and provide a contrast with previous assessments of the socio-psychological impacts of animal disease. Overall, across three different measures of subjective well-being, results fail to find consistent differences between farms with bTB and those without. Even when examining separate well-being dimensions, there appears to be little difference between well-being in bTB affected farms and those that are not. Furthermore, when considering farmers' assessments of work productivity, results again fail to find statistically significant differences between farms with and without disease. It is only when analysis focuses on areas of high bTB prevalence that there are statistically significant differences in well-being: for both the SPS and ONS measures, levels of well-being amongst farmers with bTB are ~5% lower than those without.

Why do the results reported in this paper differ from those for other exotic diseases? One reason might be that diseases such as FMD involve the imposition of emergency regulations that affect farming, its social environment and the natural environment. By contrast, the management of endemic diseases such as bTB can fail to have a clear end in sight, meaning adapting to live with disease diminishes its social impacts. The lack of relationship between well-being and bTB incidence requires further attention. Potentially, this relationship may be mediated by other factors, such as personality, the presence of other diseases and farmers' experiences of the management of bTB. Equally, this analysis does not take into account whether farms have had bTB for a long time and/or lost a large number of animals to bTB. Analysis of the relationship between well-being and other endemic diseases may confirm the findings presented here for bTB. For the moment, however, the evidence presented here suggests that any relationship between bTB and farmer well-being is complex and cannot be reduced to a simplistic conclusion.

These results have a number of implications. Firstly, by employing 3 different measures of SWB, we are able to show that assessments of the socio-psychological impacts of animal disease can depend on the precise measure used. Given the extensive testing and validation of the SWEMWBS, it is surprising that results from this scale are not reflected in results for the ONS or SPS scales. One possible explanation is that the abstract and standardized wording for some of the SWEMWBS questions (such as I've been feeling close to people) in comparison to those in the ONS or SPS scales was confusing to farmers. However, piloting of the questionnaire did not reveal any issues. Moreover, questions in all scales addressed similar issues such as feelings of optimism or the ability to deal with problems. Whilst it is not possible from these comparisons to judge which scale is most accurate, evidence from the supporting qualitative research suggests that farmers are affected by incidents of bTB on their farm. What is clear, however, is that the selection of well-being measures to be used in surveys influences results. Researchers are therefore encouraged to employ more than one measure in surveys and use qualitative research to provide methodological triangulation.

Secondly, these results may provide some support that farmers can develop coping strategies to displace the impacts of animal disease. Farmers with frequent incidents of bTB may become cynical toward Government policy and fatalistic toward preventing bTB. This lack of self-efficacy may help to mediate the effects of bTB and explain why in some cases, measures of SWB are not even lower for farmers with bTB. For example, a lack of self-efficacy may help to mediate feelings of guilt or failure. Moreover, in framing notions of well-being in relation to cultural concepts of “good farming,” the ability to blame others for failures in disease management allows farmers to retain a sense of self-worth and accomplishment. This presents a conundrum for policy makers: attempts to seek to encourage better biosecurity by aligning it with forms of “good farming” are likely to provide additional threats to farmer well-being if these measures fail. Further research is therefore required to fully understand what coping strategies farmers employ and the precise effects they have upon well-being.

Thirdly, that farmers' well-being is associated with the wider spatial epidemiological status is relevant for policy makers and organizations concerned with farmer well-being. These data suggest that those most in need of help and assistance are those farmers with bTB in high-risk areas. The extent of this relationship should be examined further using more detailed epidemiological definitions of bTB risk. In high-risk areas, farmers with bTB may benefit from talking to those without bTB to understand how they cope with the constant threat of bTB, and vice-versa. Alternatively, farmers with bTB may benefit from more targeted well-being interventions to help them manage their farm during a bTB outbreak.

Additional research is required to examine how well-being varies amongst age groups and gender. The number of young farmers in our sample is insufficient to draw any conclusions, yet there is no reason why they should not be affected by a bTB incident. Similarly, the role of farming families is worthy of further investigation to examine how other family members may be both affected by bTB and implement coping strategies (40). Finally, it is not just farmers who are involved in the management of bTB: local vets working in areas of high bTB incidence may also be affected. For these vets, bTB may affect their commitment to their profession (68) and their well-being (69, 70). As with farmers, understanding vets coping strategies to deal with the stresses of endemic disease is important for the continued management of animal disease and provision of veterinary services (71).

## Conclusion

This paper analyses the impacts of endemic disease on farmer well-being and perceived productivity. Surprisingly, results do not find a clear relationship between measures of well-being and disease incidence. For some measures, farmers with a history of disease in areas at high-risk of disease, do have lower levels of well-being. However, assessments of these impacts will likely depend on the precise well-being measures selected by researchers. As such, there is a continued need for further research, methodological discussion and debate in the study of the socio-psychological impacts of animal disease.

## Data Availability Statement

The datasets generated for this study will not be made publicly available to ensure confidentiality of respondents.

## Ethics Statement

The studies involving human participants were reviewed and approved by School of Planning and Geography Research Ethics Committee. The patients/participants provided their written informed consent to participate in this study.

## Author Contributions

DC conducted original research, analyzed data, and wrote up results. GE contributed to the design of the research methods, analyzed data, and wrote the final draft of the paper.

### Conflict of Interest

The authors declare that the research was conducted in the absence of any commercial or financial relationships that could be construed as a potential conflict of interest.
